# Orthostatic Hypotension, Frailty, and Cognitive Impairments Among Older Adults: A Hospital-based Study: Orthostatic Hypotension and Cognitive Frailty in Older Adults

**DOI:** 10.31661/gmj.v14i.3735

**Published:** 2025-07-26

**Authors:** Maryam Niksolat, Melika labaf, Vahid Rashedi, Zhale Zandieh, Hosna Mirfakhraee

**Affiliations:** ^1^ Geriatric Mental Health Research Center, Department of Geriatric Medicine, School of Medicine, Firoozabadi Clinical and Research Development Unit, Iran University of Medical Sciences, Tehran, Iran; ^2^ Department of Geriatric Medicine, School of Medicine, Iran University of Medical Sciences, Tehran, Iran; ^3^ Iranian Research Center on Aging, Department of Aging, University of Social Welfare and Rehabilitation Sciences, Tehran, Iran; ^4^ Iranian Research Center on Ageing, University of Social Welfare and Rehabilitation Sciences, Tehran, Iran; ^5^ Firoozabadi Clinical and Research Development Unit, Department of Internal Medicine, School of Medicine, Iran University of Medical Sciences, Tehran, Iran

**Keywords:** Orthostatic Hypotension, Frailty, Cognitive Impairment, Older Adults, Outpatient Geriatric Care

## Abstract

**Background:**

Orthostatic hypotension (OH) is a common condition, affecting roughly 20% of community-dwelling older adults and up to 25% of those in long-term care facilities. Its presence in older adults has been linked to heightened risks of frailty, cognitive decline, and increased fall rates, yet few studies have comprehensively examined these associations in outpatient settings. This study investigates the relationships between OH, frailty, and cognitive impairments in older adults attending an outpatient geriatric clinic.

**Materials and Methods:**

A cross-sectional study was conducted among 250 older adults (aged 60 years and above) visiting the Outpatient Geriatric Clinic at Firoozabadi Hospital. Cognitive function was evaluated using the Abbreviated Mental Test Score (AMTS) and Mini-Cog, while frailty status was determined through the Fried Frailty Index.

**Results:**

Participants had an average age of 70.72 ± 7.24 years, with ages ranging from 60 to 90. Of the total participants, 38.8% (97) were male, and 61.2% (153) were female. Average systolic blood pressure (SBP) was 132.04 ± 21.64 mmHg, and average diastolic blood pressure (DBP) was 79.43 ± 13.01 mmHg. Among participants, 38% (95) reported a history of falls, and 34% (85) were on multiple medications (polypharmacy). Notably, 33.6% (84) were diagnosed with frailty syndrome, and 29.2% (73) exhibited prefrailty. Additionally, 34.4% (86) of the participants were found to have cognitive impairments.

**Conclusion:**

The study highlights significant associations between orthostatic hypotension, frailty, and cognitive impairment among older adults. With a substantial proportion of participants displaying frailty, prefrailty, and cognitive impairments, these findings underscore the need for early screening and management of orthostatic hypotension in outpatient settings. Addressing OH could play a crucial role in mitigating frailty progression, preserving cognitive function, and reducing fall risk in older adults. Future research is warranted to explore intervention strategies that may improve the quality of life and functional outcomes in this vulnerable population.

## Introduction

Orthostatic hypotension (OH) is prevalent in the older adult population, affecting 20% of community-dwelling older adults and 25% of older adults residing in long-term care facilities [1]. OH plays a predictive role in various health outcomes, including fractures, falls, dementia, stroke, coronary heart disease, congestive heart failure, and mortality. Additionally, it can limit physical activity and functionality, significantly impacting the quality of life and leading to progressive functional deterioration [2][1][3]. OH is typically measured using the Active Standing Test (AST), which detects a decrease of ≥20 mmHg in systolic or ≥10 mmHg in diastolic blood pressure when an individual transitions from the supine position to an upright position after 3 minutes [4][5].

However, there are several considerations for reversing orthostatic hypotension, including addressing anemia, thyroid disorders, vitamin D deficiency, and B12 deficiencies [6][7], drinking more water [8], taking salt supplements [9][10], taking leg exercise [11], using compression garments [12][13][14], by head-up sleeping [15][16], and pharmacological measures like Fludrocortisone as a volume expander [17][18], and Midodrine as an alpha-1 adrenoceptor agonist to increase blood pressure (BP) by vasoconstriction [19][20]. Given that OH can be reversed, detecting and correcting it may also address its associated conditions. This study aims to evaluate the relationship between OH and frailty [21] as well as cognitive impairment [22].

Both frailty and cognitive impairment are debilitating conditions that negatively impact the lives of older adults and contribute to increased mortality. This study aims to investigate the intersection between orthostatic hypotension, frailty, and cognitive impairment among older adults attending an Outpatient Geriatric Clinic. By exploring these interrelationships, the study seeks to identify whether OH acts as both a predictor and exacerbator of frailty and cognitive decline. This research is critical in developing strategies to improve the health and well-being of older adults, as it could inform clinical interventions that target these interrelated conditions. In particular, it could lead to better management of orthostatic hypotension, potentially preventing or mitigating the negative effects on both physical and cognitive health in aging populations. Early identification and treatment of OH, particularly in frail and cognitively impaired individuals, may serve as an effective means of reducing the risk of falls, improving cognitive outcomes, and enhancing the overall quality of life for older adults.

## Material and Methods

A cross-sectional study was conducted on 250 patients aged 60 years and above who presented to the Outpatient Geriatric Clinic of Firoozabadi Hospital, affiliated with Iran University of Medical Sciences. The sample size of 250 participants was determined based on previous studies and calculated using GPower software . Key considerations included the research variables, an effect size of 0.21, an alpha level (α) of 0.05, a power (1-β) of 0.95, and an anticipated 10% dropout rate. Participants were randomly selected to enter the study, and written informed consent was obtained from all of them. Exclusion criteria included individuals who could not tolerate an upright position (sitting or standing) for 3 minutes. Participants completed a questionnaire that covered demographic information (age and gender), medical history (diabetes mellitus, hypertension, ischemic heart disease, stroke), polypharmacy (taking more than 4 medications), and a history of falling in the past year (with either serious or minor injuries). Cognitive impairment was assessed using the Abbreviated Mental Test Score (AMTS) [24], Mini-Cog [24], and Fried questionnaire for frailty [25].

The Abbreviated Mental Test Score (AMTS) is a 10-point assessment introduced by Hodkinson in 1972, designed for the rapid evaluation of older patients for potential dementia. However, it should be noted that further and more formal tests are necessary to confirm a diagnosis of dementia, delirium, or other cognitive impairments. Foroughan et al. validated and tested the reliability of this questionnaire among Persian-speaking older individuals, demonstrating that a score of 6-7 or lower indicates cognitive impairment at the time of testing.

The Mini-Cog is a quick and efficient tool for screening cognitive impairment in older adults across various healthcare settings. It is designed to rapidly detect cognitive impairment during both routine visits and hospitalizations.

The Mini-Cog comprises two components: a memory task that involves recalling three words and an evaluation of a clock-drawing task. Rezaei and colleagues tested the validity and reliability of this tool among Persian-speaking older individuals. Their findings demonstrated that the Persian version of the Mini-Cog has acceptable sensitivity, specificity, and a substantial overall agreement with the AMTS.

Fried et al. developed the Fried Frailty Scale, which includes five criteria: weight loss, exhaustion, low physical activity, slowness, and weakness. This scale is designed to identify frailty in older individuals. As the first and most widely used assessment tool for defining physical frailty, the Fried Frailty Scale has demonstrated its effectiveness as a predictor of mobility limitations and mortality.

Patients were instructed to lie down for 5 minutes, during which their blood pressures were measured. Subsequently, they were asked to either sit or stand for 3 minutes. At the end of this period, blood pressure measurements were taken again. All data were entered into SPSS 22 (Armonk, NY: IBM Corp.), and a P-value of ≤0.05 was considered statistically significant.

### Ethics Approval and Consent to Participate

This study was approved by the Ethics Committee of the Iran University of Medical Sciences (IR.IUMS.FMD.REC.1401.276) and was conducted according to the principles of the Declaration of Helsinki. Informed consent was obtained from all individual participants included in the study.

## Results

The average age of the study participants was 70.72 ± 7.24 years, ranging from 60 to 90 years. Among the cases, 38.8% (97) were male, and 61.2% (153) were female. The mean systolic blood pressure (SBP) was 132.04 ± 21.64 mmHg, with a range of 78-220 mmHg, while the mean diastolic pressure (DBP) was 79.43 ± 13.01 mmHg, ranging from 40 to 132 mmHg. Additionally, the mean heart rate (HR) was 75.84 ± 8.11 beats per minute, with a range of 54-120 beats per minute.

Among the cases, 38% (95) had a history of falling, and 34% (85) reported polypharmacy. Furthermore, 33.6% (84) exhibited frailty syndrome, and 29.2% (73) had prefrailty syndrome. Lastly, 34.4% (86) were diagnosed with cognitive impairments.

Hypotension was found in 16.8% of the participants (42 individuals). The results indicate a significant statistical correlation between hypotension and a history of falls (P-value=0.021), impaired ability to perform daily living activities at home (P-value=0.021), and cognitive impairment (P-value=0.001). No significant statistical differences were found for other variables studied (P-value>0.05).

## Discussion

This study examined the associations between OH, frailty, and cognitive impairments among older adults attending an Outpatient Geriatric Clinic. Our cross-sectional analysis demonstrated significant links between OH and various health outcomes, highlighting the complex interactions between OH, medication use, cognitive decline, and fall risk in older adults.

The prevalence of OH in our study was found to be 16%, contrasting with higher rates reported in previous research. For instance, Poon et al. identified a 55% prevalence of OH among elderly patients with cardiovascular conditions, indicating the influence of study populations and medical histories on the prevalence rate and emphasizes the significant role that comorbidities, particularly cardiovascular conditions, and polypharmacy play in exacerbating OH risk [26]. Polypharmacy, especially with antihypertensive and psychoactive medications, can increase the likelihood of postural hypotension, pointing to the importance of careful medication management in older adults.

We observed a positive correlation between a decrease in blood pressure upon standing and both polypharmacy and cognitive impairment. This supports findings from earlier studies, such as a 2010 cross-sectional study that emphasized the importance of managing medication regimens to mitigate OH effects [27]. Reducing the use of drugs that lower blood pressure, may help prevent OH and its complications, especially in those at risk for cognitive decline.

The relationship between OH and fall risk remains intricate and multifaceted. While a prospective cohort study from 2021 found that improving diastolic blood pressure soon after standing was linked to a lower risk of falls [28], our study did not find a direct association between OH and past falls. This is consistent with findings from a 2015 cross-sectional analysis, which also did not find a strong link between OH and fall history [29]. This discrepancy in the timing and recovery rate of blood pressure changes may be critical in assessing fall risk, as OH could lead to momentary dizziness that increases the chance of falling, especially in frail individuals.

Our study also identified a significant association between OH and cognitive impairments, a finding that aligns with several recent studies. For example, a 2021 longitudinal cohort study found that persistent OH was associated with cognitive decline in patients with Alzheimer’s disease [30]. However, not all studies point to the complexity of the relationship between OH and cognitive function. Cerebral hypoperfusion—a reduced blood supply to the brain resulting from postural hypotension—may contribute to cognitive decline by depriving the brain of adequate oxygenation. This hypothesis is bolstered by research showing that chronic OH can impair cerebral autoregulation, potentially leading to structural brain changes over time.

These mixed findings suggest that multiple mechanisms, including both physiological factors and medication effects, are likely at play in the relationship between OH, cognitive impairment, and fall risk. Future research could benefit from a longitudinal approach, investigating the effects of OH on cognitive outcomes over time while considering the duration and intensity of blood pressure drops after standing. Additionally, examining the role of polypharmacy in more depth could clarify how specific drug classes impact OH and cognitive function.

### Clinical Implications and Recommendations

Given the potential adverse effects of OH on falls, fractures, and cognitive decline—particularly in older populations—there are key areas for intervention that can help improve patient outcomes and reduce associated health risks. Addressing OH in clinical practice is essential for supporting safe aging, preventing injuries, and maintaining cognitive health. Here are several targeted strategies and recommendations:

1. Medication Review and Management: Adjusting medication regimens, especially in those using blood pressure-lowering medications or diuretics, can mitigate OH-related risks. Clinicians should consider alternatives to medications known to exacerbate OH, such as loop diuretics, or adjust dosages to reduce postural blood pressure drops. Regular medication reviews, particularly for older adults on complex regimens, are crucial in managing OH while maintaining therapeutic efficacy.

2. Promoting Physical Activity and Tailored Exercise Programs: Customized exercise programs aimed at improving balance, strength, and cardiovascular endurance can enhance orthostatic tolerance and reduce the incidence of OH. Specific exercises, such as resistance training and balance exercises, can also help strengthen postural stability, thereby minimizing fall risk. Clinical exercise physiologists and geriatricians can play an important role in creating personalized exercise regimens suitable for older adults at risk of OH.

3. Active Aging and Prolonged Workforce Participation: Encouraging active engagement in social and occupational activities may offer protective effects against the consequences of OH. Maintaining physical activity and mental engagement, even within modified work environments, can reduce sedentary behavior, promote cardiovascular health, and enhance postural stability. This active approach to aging not only promotes independence but can also lessen the burden of OH-related complications.

4. Regular OH Screening and Monitoring in Primary Care: Routine screening for OH in older adults, especially in those with known cardiovascular risk factors, frailty, or recent falls, should be incorporated into standard primary care practices. Implementing standing blood pressure measurements as part of routine vital signs in geriatric patients allows for early identification of OH and timely interventions to prevent associated risks.

5. Patient Education and Self-Management Strategies: Educating older adults and their caregivers about OH and strategies to manage symptoms—such as rising slowly, staying hydrated, and wearing compression stockings—can empower them to manage their condition effectively. Self-management techniques are particularly beneficial for enhancing daily functioning and reducing the risk of adverse events associated with OH.

### Limitations and Future Work

Our study had several limitations that should be taken into account when interpreting the findings. The limited timeframe for participant recruitment, coupled with the study being conducted at a single hospital and center, may have influenced the representativeness of our study population. As a result, this could potentially impact the generalizability of our findings. The demographic and health characteristics captured during this period might not fully reflect the broader elderly population in the region. Future research with an extended sampling period would help ensure a more diverse and representative sample, thereby improving external validity. Although our study yielded important insights, the relatively small sample size may have restricted our ability to fully explore the relationships between orthostatic hypotension and various hypothesized risk factors, including ethnicity, socioeconomic background, and lifestyle factors. Expanding the sample size in future studies would enhance statistical power, allowing for more detailed subgroup analyses and more reliable conclusions. Some associations observed between orthostatic hypotension and specific factors—such as the use of mobility aids and lower activity levels—may reflect outcomes of orthostatic hypotension rather than underlying causes. For instance, individuals experiencing frequent dizziness or instability may naturally limit their mobility, which could lead to further health decline. Future studies could incorporate longitudinal designs to better differentiate between causal and consequential relationships, providing a clearer picture of risk factors and their effects. Differences in measurement protocols, such as the timing of blood pressure assessments and distinctions between systolic and diastolic changes, may contribute to discrepancies in prevalence rates and associations. Standardizing assessment protocols for orthostatic hypotension across research studies would help ensure consistency, allowing for more accurate comparisons and insights into the condition’s effects on health.

Despite these limitations, this study offers valuable preliminary data that can guide future research. Further large-scale, longitudinal studies with rigorous sampling and standardized assessments are essential to clarify orthostatic hypotension's risk factors and consequences. Additionally, identifying modifiable risk factors could inform the development of targeted interventions aimed at reducing fall risk, preventing cognitive decline, and improving quality of life among older adults.

## Conclusion

The presence of OH occurring within 3 minutes of assuming an upright position is strongly linked with both a heightened risk of falls within the subsequent year and cognitive impairment in older adults. This relationship underscores the need for vigilant monitoring of blood pressure changes in this population, as even subtle drops in systolic or diastolic pressure can indicate underlying vulnerabilities. Variations in study findings on OH, however, highlight a challenge: inconsistencies in how OH is defined and measured, including differences in assessing systolic versus diastolic drops and the protocols used to evaluate blood pressure changes. This heterogeneity suggests that a standardized approach to diagnosing OH may be crucial for enhancing the reliability and applicability of research results across diverse populations.

Ultimately, our findings support the critical role of routine screening and targeted interventions to manage OH in older adults, potentially reducing fall risk and preserving cognitive function. Future studies should seek to clarify the impact of specific blood pressure thresholds and the dynamics of both systolic and diastolic OH concerning frailty and cognitive decline. Addressing these factors could guide more personalized clinical interventions, aiming not only to improve safety and functional independence but also to support the overall quality of life in older adults.

## Conflict of Interests

None.
